# Inter-rater reliability for assessing intracranial collaterals in patients with acute ischemic stroke: comparing 29 raters and an artificial intelligence-based software

**DOI:** 10.1007/s00234-022-02984-z

**Published:** 2022-05-24

**Authors:** Lennard Wolff, Jiahang Su, Derek Van Loon, Adriaan van Es, Pieter Jan van Doormaal, Charles Majoie, Wim van Zwam, Diederik Dippel, Aad van der Lugt, Theo van Walsum, on behalf of the MR CLEAN investigators

**Affiliations:** 1 Department of Radiology and Nuclear Medicine, Erasmus MC University Medical Center, Doctor Molewaterplein 40, 3015 GD Rotterdam, The Netherlands; 2 Department of Radiology, Leiden University Medical Center, Leiden, The Netherlands; 3 Department of Radiology & Nuclear Medicine, Amsterdam University Medical Centers, location AMC, Amsterdam, the Netherlands; 4 Department of Radiology and Nuclear Medicine, Maastricht University Medical Center, Maastricht, The Netherlands; 5 Department of Neurology, Erasmus MC University Medical Center, Rotterdam, The Netherlands

**Keywords:** Ischemic stroke, Collateral circulation, Reproducibility of results, Algorithms, Consensus

## Abstract

**Purpose:**

Outcome of endovascular treatment in acute ischemic stroke patients is depending on the collateral circulation maintaining blood flow to the ischemic territory. We evaluated the inter-rater reliability and accuracy of raters and an automated algorithm for assessing the collateral score (CS, range: 0–3) in acute ischemic stroke patients.

**Methods:**

Baseline CTA scans with an intracranial anterior occlusion from the MR CLEAN study (*n*=500) were used. For each core lab CS, ten CTA scans with sufficient quality were randomly selected. After a training session in collateral scoring, all selected CTA scans were individually evaluated for a visual CS by three groups: 7 radiologists, 13 junior and 9 senior radiology residents. Two additional radiologists scored CS to be used as reference, with a third providing a CS to produce a 2 out of 3 consensus CS in case of disagreement. An automated algorithm was also used to compute CS. Inter-rater agreement was reported with intraclass correlation coefficient (ICC). Accuracy of visual and automated CS were calculated.

**Results:**

39 CTA scans were assessed (1 corrupt CTA-scan excluded). All groups showed a moderate ICC (0.689-0.780) in comparison to the reference standard. Overall human accuracy was 65± 7% and increased to 88± 5% for dichotomized CS (0–1, 2–3). Automated CS accuracy was 62%, and 90% for dichotomized CS. No significant difference in accuracy was found between groups with different levels of expertise.

**Conclusion:**

After training, inter-rater reliability in collateral scoring was not influenced by experience. Automated CS performs similar to residents and radiologists in determining a collateral score.

**Supplementary Information:**

The online version contains supplementary material available at 10.1007/s00234-022-02984-z.

## Introduction

Acute ischemic stroke is mainly caused by the occlusion of one or more brain arteries, which leads to an inadequate supply of oxygen to a region of the brain [1]. Globally, ischemic stroke is the second leading cause of death, and a major contributor to disability-adjusted life years in the population [2]. The most effective treatment in ischemic stroke is timely reperfusion of the occluded arteries [3]. Patients with an intracranial large vessel occlusion could be eligible for endovascular thrombectomy (EVT). The thrombus is thereby removed from the vessel with a stent retriever and/or aspiration device, restoring the original blood flow and oxygen supply [4]. However, EVT is not an intervention without risks, and the effect of treatment will vary between individuals [5]. The effect of EVT is dependent on different clinical and imaging parameters. These parameters can be assessed pre-operatively to determine the chance of a treatment benefit and for patient selection, especially in the late time window [6]. Therefore, it is important to investigate how consistent and reliable these parameters can be obtained pre-operatively.

One of the parameters relevant to determine treatment effect is the collateral score (CS) [7]. The CS quantifies the contrast filling of the distal MCA branches through the arterial collateral circulation in the affected hemisphere. The collaterals are secondary pathways which can function as a back-up when the primary arteries fail to deliver an adequate blood supply [8]. Brain tissue at risk due to an occlusion is more likely to survive a period with insufficient blood supply through primary pathways if oxygen supply is ensured through collateral vessels [8].

Tan et al. developed a 4-point categorical grading system for assessment of collateral status in the occluded middle cerebral artery (MCA) territory on a computed tomography angiography (CTA) scan [9]. A score of 0 is given for absent collaterals, 1 for > 0% and ≤ 50% collateral supply filling, 2 for > 50%, and 3 for 100% filling of the occluded MCA territory [9]. The collateral score for a CTA scan is generally obtained by visual scoring, which is operator dependent with potential interobserver variation. Machine learning-based approaches to produce an automated quantitative collateral score (qCS) showed similar performance to that of experienced radiologists [10].

In this study, we aim to assess the interobserver variability for the CS and whether variability is influenced by years of experience [11]. Secondly, we compare the visual CS given by physicians with the previously mentioned qCS and a reference CS.

## Methods

### Imaging data

Baseline CTA scans were acquired from the Multicenter Randomized Clinical Trial of Endovascular Treatment for Acute Ischemic Stroke in The Netherlands (MR CLEAN, MR CLEAN Netherlands Trial Registry number: NTR1804. Current Controlled Trials number, ISRCTN10888758), a prospective, consecutive study which was performed in 16 stroke centers in the Netherlands [6]. The MR CLEAN study protocol was approved by the central medical ethics committee of the Erasmus MC and the research board of each participating center. All patients or their legal representatives provided written informed consent before randomization.

The MR CLEAN database contains data from 500 patients with acute ischemic stroke caused by an occlusion in the anterior circulation. Pre-interventional CTA scans were rated for CS and occlusion location by a core lab of radiologists without access to other imaging data or any clinical information. CTA scans with good/moderate image quality; adequate head coverage; axial series; slice thickness <1.0 millimetres; and slice increment equal to or smaller than slice thickness were selected. From those CTA scans, ten scans were randomly selected for each CS. Axial and coronal maximum intensity projections (MIPs) with slice thickness of 8 millimetres were reconstructed.

### Visual collateral scoring

If applicable, year of residency was recorded. Seven radiologists and 22 radiology residents in the Netherlands attended a 1-h training session in collateral scoring. The rationale and method of scoring were explained, and CS examples were shown and discussed.

After the training, attendees were asked to score all cases based on the axial CTA scan and 8 mm MIP reconstructions combined. The occluded vessel (ICA-T/M1/M2) and affected hemisphere (left/right) were given for each case.

### Automated collateral scoring

qCS were produced with the model reported by Su et al. [10]. CTAs acquired in the MR CLEAN study were not used for the development of this model. Processing of the CTA scan started with an atlas-based registration and segmentation of the vessel centrelines using a neural network. After this, the relative amount of vessels in the MCA territory was quantified by comparing the affected hemisphere with the unaffected hemisphere in terms of vessel volume and vessel length, both weighted and unweighted for pixel intensity. The four ratios were used to compute a qCS. The qCS was converted to a collateral score using a modified definition of Tan et al.: collateral score 0 was defined as equal or less than 10% filling instead of 0% filling of the affected MCA territory [9].

### Reference standard

The CTAs which were evaluated by the imaging core lab were re-evaluated for CS by two independent and experienced interventional neuroradiologists (A.v.E, P.J.v.D), who were not part of the group of raters. In case of disagreement, the core lab observer rating was used as third CS to provide a two-observer consensus.

### Statistical analysis

The results were analysed after grouping the respondents as follows: first and second-year radiology residents (junior residents, *n*=13), radiology residents in years 3–5 (senior residents, *n*=9), all radiology residents (*n*=22), radiologists (*n*=7), and all physicians combined (*n*=29). The mean and standard deviation of the visual CS were calculated. Analysis was performed on the 4-point collateral score and on dichotomized assessments (CS 0–1: poor collaterals; CS 2–3: good collaterals). Dichotomisation was performed since treatment effects in MR CLEAN patients with good collaterals were substantial, whereas treatment effects were small in patients with poor collaterals [7].

Observer variability was reported using an intraclass correlation coefficient (ICC) using a two-way random, single measures, absolute agreement model [12]. ICC was calculated for the 4-point collateral score and a dichotomized score for all observers and for subgroups based on experience in radiological readings: junior residents, senior residents and radiologists. An ICC below 0.50 indicates poor, > 0.50 and ≤ 0.75 moderate, > 0.75 and ≤ 0.90 good, and > 0.90 excellent correlation. Accuracy for full CS and dichotomized CS was calculated for each group compared to the reference standard and qCS. Significant differences were calculated using One-way ANOVA. Statistical analyses were performed using SPSS Statistics Version 25.

## Results

### Selection of dataset and reference

**Fig. 1:**
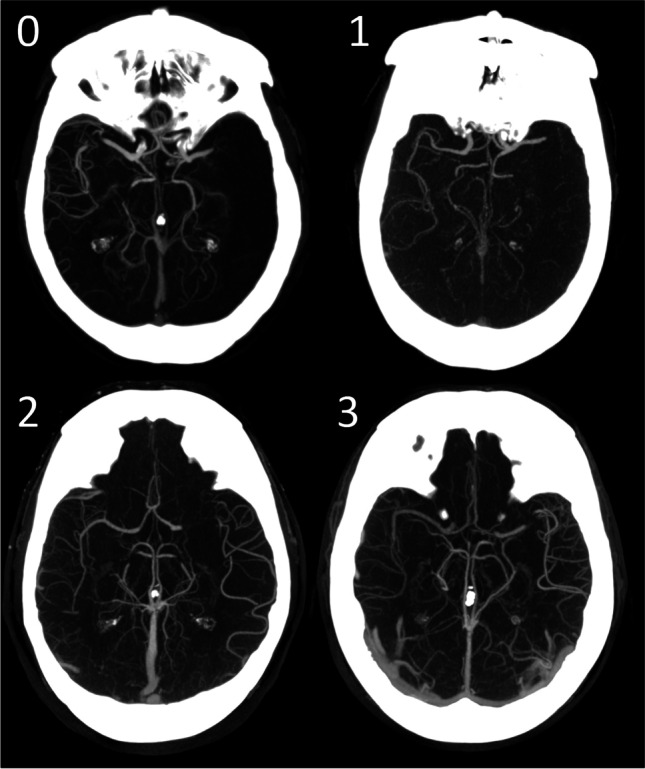
Visual collateral score grading in patients with an M1 occlusion. 0—absent collaterals, 0% filling of the occluded territory. 1—poor collaterals, >0% and ≤50% filling of the occluded territory. 2—moderate collaterals, >50% and <100% filling of the occluded territory. 3—good collaterals, 100% filling of the occluded territory

**Fig. 2:**
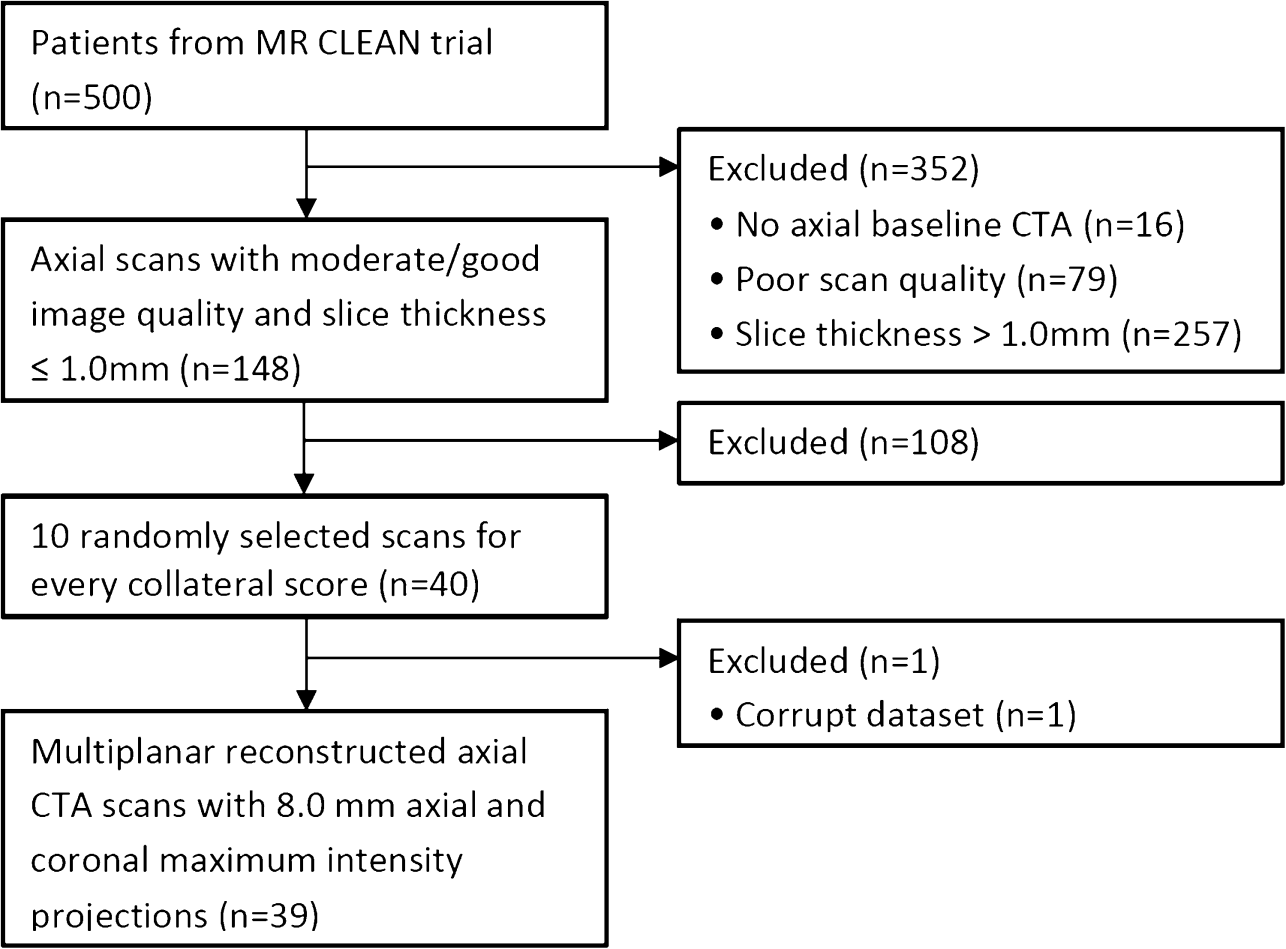
Selection of CTA scans. CTA, computed tomography angiography; mm, millimetres

From the 500 patients, 148 patients met inclusion criteria. For CS 0–3 (Fig. 1), based on image core lab evaluation, 10 CTA scans were randomly selected per collateral score. One scan could not be processed to create MIPs, which resulted in a test set with 39 cases. Figure 2 shows a schematic visualization of the patient selection. For the 39 selected cases, a reference CS was obtained after rereading the scans: CS 0 (*n*=5), CS 1 (*n*=13), CS 2 (*n* = 10), and CS 3 (*n* = 11) (Table 1). In 31% of the cases (12/39), a third radiologist was needed to provide consensus. CTA scan details (reference collateral score, slice thickness, peak kilovoltage (kVp), exposure (mA)) were reported (supplementary Table 1).

**Table 1:** Reference collateral score and occlusion location

Reference collateral score	Occlusion location			Total
	ICA-T	M1	M2	
0	1	4	0	5
1	5	7	1	13
2	5	3	2	10
3	3	7	1	11
Total	14	21	4	39

### Inter-rater variability

The ICC is reported for the 4-point CS (Table 2) and for dichotomized CS (Table 3). All groups showed a moderate to good ICC with an ICC of 0.751 (95% CI: 0.665–0.835) for the combined results (Table 2). When dichotomizing CS, ICC for all observers combined decreased to 0.682 (95% CI: 0.585–0.783) (Table 3). No differences in ICC were demonstrated between the subgroups.

**Table 2:** Correlation with reference collateral score for physician groups

Group (*n*)	4-point scale collateral score, intraclass correlation coefficient (95% CI)		
1^st^ year residents (10)	0.780 (0.696–0.857)	0.775 (0.693–0.853)	0.751 (0.665–0.835)
2^nd^ year residents (3)			
3^rd^ year residents (4)	0.766 (0.676–0.849)		
4^th^ year residents (4)			
5^th^ year residents (1)			
Radiologists (7)	0.689 (0.577–0.795)

**Table 3:** Correlation with dichotomized reference collateral score for physician groups

Group (*n*)	Dichotomized collateral score^a^, intraclass correlation coefficient (95% CI)		
1^st^ year residents (10)	0.722 (0.626–0.816)	0.698 (0.602–0.796)	0.682 (0.585–0.783)
2^nd^ year residents (3)			
3^rd^ year residents (4)	0.675 (0.566–0.783)		
4^th^ year residents (4)			
5^th^ year residents (1)			
Radiologists (7)	0.636 (0.516–0.755)		

^a^Dichotomized in poor (collateral score 0–1) and good (collateral score 2–3) collateral status.

### Accuracy

The mean accuracy for rating CS by the 29 raters was 65± 7%, (Table 4). No significant differences in accuracy were found between the subgroups (Table 4). Accuracy increased to 88± 5% when a dichotomized scale was used; however, the differences between subgroups remained statistically insignificant (Table 5). When using qCS (categorized, 0–3) as reference score, the mean overall accuracy was 67 ± 8%, which increased to 88 ± 5%, after dichotomization (CS 0–1; poor collaterals, CS 2–3; good collaterals). The accuracy for scoring CS was 62% for qCS, which increased to 90% after dichotomization of CS.

**Table 4:** Collateral score accuracy for physician groups

Group (*n*)	4-point scale collateral score, accuracy (standard deviation)*		
1^st^ year residents (10)	62± 8%	64± 7%	65± 7%
2^nd^ year residents (3)			
3^rd^ year residents (4)	66± 7%		
4^th^ year residents (4)			
5^th^ year residents (1)			
Radiologists (7)	67±7%		
qCS (1)	62% (n.a.)		

*All differences in accuracy were not statistically significant with *p* > 0.05

**Table 5:** Dichotomized collateral score accuracy for physician groups

Group (*n*)	Dichotomized collateral score^a^, accuracy (standard deviation)*		
1^st^ year residents (10)	87± 5%	87± 5%	88± 5%
2^nd^ year residents (3)			
3^rd^ year residents (4)	89± 4%		
4^th^ year residents (4)			
5^th^ year residents (1)			
Radiologists (7)	88± 4%		
qCS (1)	90% (n.a.)		

^a^Dichotomized in poor (collateral score 0–1) and good (collateral score 2–3) collateral status. *All differences in accuracy were not statistically significant with *p* > 0.05

### Individual case analysis

**Fig. 3:**
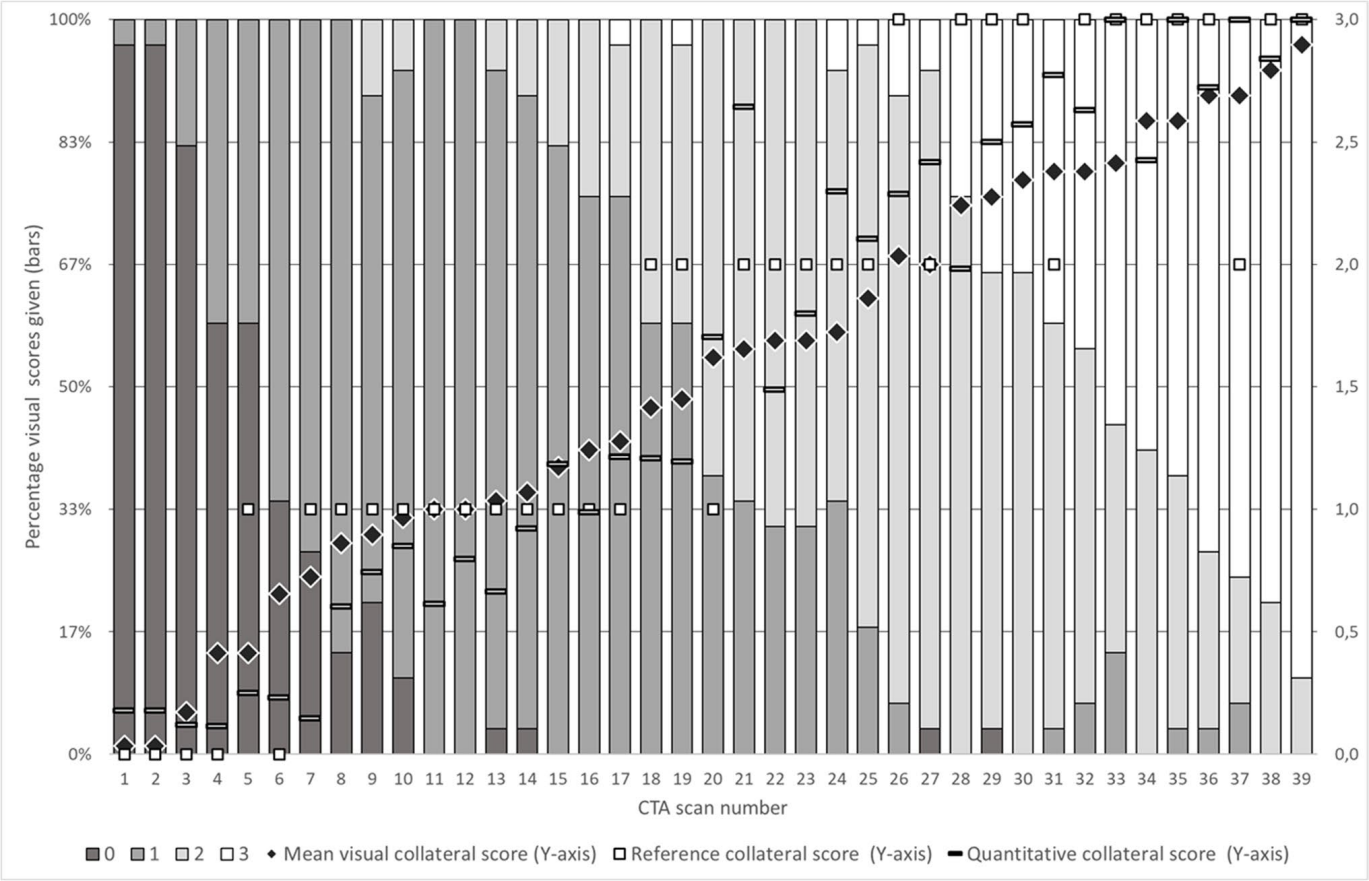
Collateral scores separated by case and ranked on average collateral score.  Black vertical bars: visual collateral score 0.  Dark grey vertical bars: visual collateral score 1.  Grey vertical bars: visual collateral score 2.  Light grey vertical bars: collateral score 3.  Black tilted square: mean visual score.  White box: reference score.  White bar: quantitative score

The mean visual CS (range: 0–3) per case ranged from 0.03 to 2.90. Full agreement in visual CS occurred in 2 cases (5%). Observers appointed 2 different CS in 20 cases (51%) and 3 different CS in the remaining 17 cases (44%). The qCS (range: 0–100%) ranged from 3.79 to 100%. Individual cases were sorted by ascending mean visual CS and visualized in Fig. 3.

## Discussion

In this study, we evaluated the observer variability for visual collateral scoring and compared scores given by respondents after a 1-h training session and scores from automated software with reference scores. No difference was found between the different radiology resident groups and radiologists for performance in scoring CS. Accuracy in comparison with the reference was similar for all groups. Automated CS performs similar to residents and radiologists in determining a collateral score.

The inter-rater variability of scoring collateral circulation status has been reported before, but often this is done with Cohen’s kappa [7, 13, 14]. However, using Cohen’s kappa for a not-dichotomized score is harder to interpret because the differences between scores must be weighted based on the distance between categories, which happens when using weighted Kappa or ICC. In a study by Weiss et al., weighted Kappa was provided for inter-rater reliability in scoring collateral status, but they used only 2 readers [15]. An ICC of 0.87 for 4-point CS was given by Tan et al., but this was also based on 2 readers [9]. Using more observers would be preferable when assessing inter-rater reliability.

The automated CS shows comparable accuracy with visual raters for both the 4-point CS and the dichotomized score. There are other algorithms reported in literature for automatic CS. The research from Boers et al. presents a quantitative model which calculates the percentage of vascular presence of the occluded territory in comparison to the unaffected hemisphere [16]. A different approach was used for vessel recognition. It also showed a significant correlation (Spearman *ρ*: 0.75, *P* < .001) with the categorical visual CS (0–3) as defined by Tan et al., but an ICC, accuracy, or error matrix has not been reported [9].

Research from Grunwald et al. evaluates automated collateral score software from Brainomix Ltd. in clinical practice [17]. This software uses basic image segmentation and machine learning, both not further specified. The output is a 4-point scale collateral score. They reported an agreement of 90% and a non-specified ICC of 0.93 (95% CI 0.90–0.95) for the automated collateral score software in comparison to the reference score. However, the reference score was constructed with information on the automated collateral scores.

Collateral status can be used to predict outcome [18]. It is important to use the correct imaging to assess the collateral status for predicting outcome. Assessment of collateral status on multiphase CTA instead of single phase CTA showed a better performance in predicting outcome [19]. Optimal collateral assessment is after the peak arterial phase [20]. A limitation in the accuracy calculations is the categorical reference CS. It is difficult to reach consensus, even among experienced neuroradiologists. In 31% of the cases (12/39), a third radiologist was needed to provide consensus. Incorporating software to aid in determining collateral status can help to minimize the interobserver variability while maintaining high accuracy. For the quantitative model, the calculated CS can be given over the full range from 0 to 100% to add nuance to the score.

Radiological experience differs vastly between radiology residents and senior radiologists; however, no differences were found in accuracy of CS assessment. Based on those results, we expect other radiologists to perform similar in this setting. For the same reason, we do not expect the results from residents and radiologists to improve. Furthermore, the overall performance (ICC: 0.751, 95% CI: 0.665–0.835) is comparable to previously reported interobserver agreement for scoring collateral circulation status [7, 9, 14, 21].

We believe that achieving 65% accuracy and an ICC of 0.751 is possible for all radiology residents and radiologists after basic collateral score training. The definition of the score requires categorization of a quantitative value based on visual inspection. A difference between 90 and 20% will be clearly visible, but a difference between 45 and 55% is hard to distinguish. The difference in both situations is 1 point and questions the use of categorical scoring for collateral status. Using a quantitative scale rather than a categorical one may result in better treatment decisions. Quantitative automated CS software could be a solution, but validation is needed before integrating the software in clinical practice. The next step for validating the automatic CS would be to investigate the predictive performance: can they predict the functional outcome of patients based on the baseline scans? The modified Rankin Scale (mRS) 3 months after acute ischemic stroke is commonly used to assess functional outcome [22]. The number of investigated scans (*n*=39) limits the predictive power of this study, and therefore the feasibility of analysing predictive performance. Future research using a larger sample size is recommended to investigate the correlation between automated CS and mRS in comparison to visual CS. Ideally, a study should be conducted in which both the visual CS and the automated quantitative CS are used for the same acute ischemic stroke patients to determine the performance of those collateral scores in predicting outcome and treatment benefit. Furthermore, not only the collateral score should be taken into account, also other proven predictors for outcome and/or treatment benefit should be included, combining those in a large prediction model, such as the MR PREDICTS decision tool [23]. Then, the performance of the two CS types can be compared in the prediction model.

## Conclusions

On the individual rater level, there is considerable variability in rating collateral status. After a 1-h training, the accuracy of scored CS with a reference standard is not influenced by rater experience. Automated CS shows a similar performance as experienced radiologists and radiology residents. Automated CS can be an aid for physicians, especially for cases with borderline collateral scores.

## Supplementary information

Below is the link to the electronic supplementary material.

Supplementary file1 (XLSX 9 kb)

## Data Availability

The datasets generated during the current study are available from the corresponding author on reasonable request. The other datasets analysed during the current study are available from the MR CLEAN trial office (mrclean@erasmusmc.nl) on reasonable request.

## Code availability

The code generated during the current study is available from the corresponding author on reasonable request.
